# Graphene Oxide Covalently Functionalized with 5-Methyl-1,3,4-thiadiazol-2-amine for pH-Sensitive Ga^3+^ Recovery in Aqueous Solutions

**DOI:** 10.3390/molecules29163768

**Published:** 2024-08-09

**Authors:** Xi Zhu, Yong Guo, Baozhan Zheng

**Affiliations:** College of Chemistry, Sichuan University, Chengdu 610065, China; 265cici@163.com (X.Z.); zhengbaozhan@scu.edu.cn (B.Z.)

**Keywords:** graphene oxide, composite, adsorption, selectivity, gallium

## Abstract

A novel graphene-based composite, 5-methyl-1,3,4-thiadiazol-2-amine (MTA) covalently functionalized graphene oxide (GO-MTA), was rationally developed and used for the selective sorption of Ga^3+^ from aqueous solutions, showing a higher adsorption capacity (48.20 mg g^−1^) toward Ga^3+^ than In^3+^ (15.41 mg g^−1^) and Sc^3+^ (~0 mg g^−1^). The adsorption experiment’s parameters, such as the contact time, temperature, initial Ga^3+^ concentration, solution pH, and desorption solvent, were investigated. Under optimized conditions, the GO-MTA composite displayed the highest adsorption capacity of 55.6 mg g^−1^ toward Ga^3+^. Moreover, a possible adsorption mechanism was proposed using various characterization methods, including scanning electron microscopy (SEM) equipped with X-ray energy-dispersive spectroscopy (EDS), elemental mapping analysis, Fourier transform infrared (FT-IR) spectroscopy, and X-ray photoelectron spectroscopy (XPS). Ga^3+^ adsorption with the GO-MTA composite could be better described by the linear pseudo-second-order kinetic model (*R*^2^ = 0.962), suggesting that the rate-limiting step may be chemical sorption or chemisorption through the sharing or exchange of electrons between the adsorbent and the adsorbate. Importantly, the calculated *q_e_* value (55.066 mg g^−1^) is closer to the experimental result (55.60 mg g^−1^). The well-fitted linear Langmuir isothermal model (*R*^2^ = 0.972~0.997) confirmed that an interfacial monolayer and cooperative adsorption occur on a heterogeneous surface. The results showed that the GO-MTA composite might be a potential adsorbent for the enrichment and/or separation of Ga^3+^ at low or ultra-low concentrations in aqueous solutions.

## 1. Introduction

The extraction of vanadium from vanadium titano-magnetite ores by salt roasting is one of the most representative routes. After sodium vanadate is leached out, several rare, dispersed elements, such as indium (In), gallium (Ga), and scandium (Sc) at low concentration levels, remain in the vanadium slag-processing residue (VSPR), causing undesirable environmental risks and resource waste [1], receiving considerable attention from the Ministry of Ecology and Environment. Ga is one of the most important strategic materials for high-tech applications, playing an irreplaceable role in the electronic and aerospace industries. However, Ga-containing resources are unevenly distributed globally, and their presence cannot be guaranteed [2]. In addition, the low Ga content in minerals makes it extremely difficult to guarantee its economically feasible recovery. To recover Ga, In, and Sc at low concentration levels in aqueous solutions, various technologies have been developed. Fortunately, the enrichment of Ga seems possible through adsorption treatment technologies [3]. Thus, the development of effective adsorbents for the highly efficient and selective recovery of Ga is urgent and worthy of investigation.

Adsorption is thought to be one of the most efficient and convenient strategies, but its mechanism is still unclear [2,4]. The adsorption of Ga^3+^ and In^3+^ by γ-Al_2_O_3_ suggests that bidentate Ga^3+^ and In^3+^ and monodentate GaOH^2+^/InOH^2+^ might be formed on the surface, benefiting efficient adsorption [5]. The adsorption of Ga^3+^ from aqueous solutions by oxidized coir (OC) was implemented in another study, and a high adsorption rate of 70.53% and an adsorption capacity of 19.42 mg g^−1^ could be obtained at pH = 3 in the pH range of 1~3 due to the ion exchange interactions [6]. The pH and the adsorbent dosage were found to have a positive effect on the adsorption of Ga^3+^ by bentonite, indicating that the former might be an important and significant factor [7]. The cationic or anionic contaminants adsorbed on the surfaces of an adsorbent material are usually dominated by a specific mode of adsorption—chemisorption [8]. That is, ion exchange, electrostatic interactions, and an electro-donor/acceptor (EDA) might be involved in the adsorption process. The solution’s pH values greatly affect the adsorbent’s surface charges and the hydrolysis of the adsorbate; therefore, optimal adsorption is usually favored at a specific pH value, which is close to the acid dissociation constant (pKa). Additionally, the intrinsic surface complexation constant (*K_C_*_,*intr*_) between the positive/negative ions and the adsorbent is also very important [9]. To develop a highly efficient and selective adsorbent, the rational design of its structure and the grafted functional groups are important, as these are undoubtedly dominant in influencing its adsorption performance.

Graphene oxide (GO)-based composites have recently attracted great attention in the field of adsorption and molecular separation [10]. The modification of GO with different organic molecules could endow it with specific adsorption properties, such as high efficiency, good selectivity, and excellent reusability [11,12,13]. Specifically, the adsorbability of GO-based composites toward heavy metal ions, rare earth elementals (REEs), or organic contaminants (including dyes, phenols, antibiotics, and pesticides) could be regulated [14,15,16]. Previous research has suggested that nitrogen (N)-containing heterocyclic compounds could be used to modify GO to selectively adsorb specific metal ions in aqueous solutions [17,18]. Evidently, the existence of C=N and -NH_2_ groups in 2-aminobenzothiazole (ABT) would benefit the selective adsorption of REEs [17], while N-enriched lignosulfonate could selectively interact with hexavalent chromium (Cr^6+^) [18]. Previous research has also suggested that 2-mercapto-5-methyl-1,3,4-thiadiazole and 2,5-bis(methylmercapto)-1,3,4-thiadiazole could be combined with Ni^2+^, Cu^2+^, Zn^2+^, Cd^2+^, and Hg^2+^ [19,20], revealing that the different bonding modes of N-containing groups produce quite different adsorption characteristics or affinities.

In another study, the highly effective adsorption of Ga^3+^ from waste Bayer solution was successfully carried out using polyacrylonitrile nanofiber membranes (PAN NFs) containing amidoxime groups [21], suggesting that certain N-containing groups could selectively interact with Ga^3+^. There have been few reports so far on the evaluation of N-containing molecules with modified GO for Ga^3+^ adsorption, and it is of great significance to investigate novel GO-based composites for the highly efficient and selective enrichment of Ga^3+^ in aqueous solutions. The construction of Ga^3+^-imprinted polyacrylic acid on GO (IIP-GO/PAA) using controllable polymerization and surface-imprinting technology has previously been implemented; this exhibited a better adsorption capacity (221.56 mg g^−1^) for Ga^3+^ and higher selectivity in comparison with Al^3+^, Fe^3+^, Mg^2+^, and Ca^2+^ in the acid-leaching process of fly ash [22]. However, some shortcomings, such as the complicated and uncontrollable operations, were also apparent. The selective adsorption of Ga^3+^ in the presence of Sc^3+^ and In^3+^ seems to be more difficult because their properties are very similar. Previously, a 4-amino-3-hydrazino-1,2,4-triazol-5-thiol (AHTZT)-modified GO (GO-AHTZT) composite, possessing a relatively lower adsorption capacity and an unremarkable selectivity for Ga^3+^, was developed; this process was preferentially protonated due to the existence of free amino and hydrazine groups, causing inevitable electrostatic repulsions with Ga^3+^ [23]. 

In this work, to improve the adsorption capacity and selectivity of GO-based materials, an interesting organic ligand, 5-methyl-1,3,4-thiadiazol-2-amine (MTA), was covalently coupled with GO to produce a novel composite through nucleophilic addition. We proposed that it might possess adsorption selectivity for Ga^3+^ due to the heterocyclic -C=N-N=C- groups. Because of the basic properties of the heterocyclic -C=N-N=C- and -NH- groups, the GO-MTA composite might also be pH-sensitive. Batch adsorption experiments were performed to evaluate the adsorbability of a GO-MTA composite for Ga^3+^, and the adsorption mechanism was proposed by fitting the experimental data with different adsorption kinetic and isotherm models.

## 2. Results and Discussion

### 2.1. Characterization Results

#### 2.1.1. SEM, EDS, and Elemental Mapping Analyses

**Figure 1:**
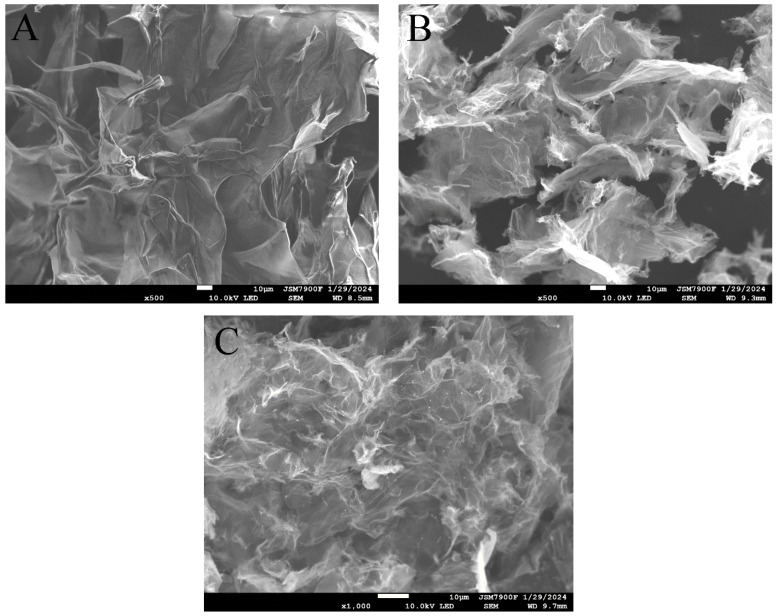
SEM images of the samples: (**A**) GO; (**B**) GO-MTA composite; and (**C**) GO-MTA composite post Ga^3+^ adsorption.

The surface morphologies of the fabricated samples were recorded via SEM (Figure 1). Lamellar GO structures could be clearly observed, indicating that the chemical exfoliation of the flake graphite had been successfully performed (Figure 1A). Many wrinkles near the edges of the GO sheets could also be found, suggesting that single- or multi-layered lamellar structures can provide abundant active sites for further modification [24,25]. After the chemical attachment of MTA, the prepared GO-MTA composite exhibited a thickened multi-layered structure with a rougher surface due to the improved π-π stacking and hydrogen bonding interactions (Figure 1B). After Ga^3+^ adsorption, the morphology of the GO-MTA composite remained almost unchanged, confirming the structural stability of the developed GO-based adsorbent (Figure 1C). 

**Figure 2:**
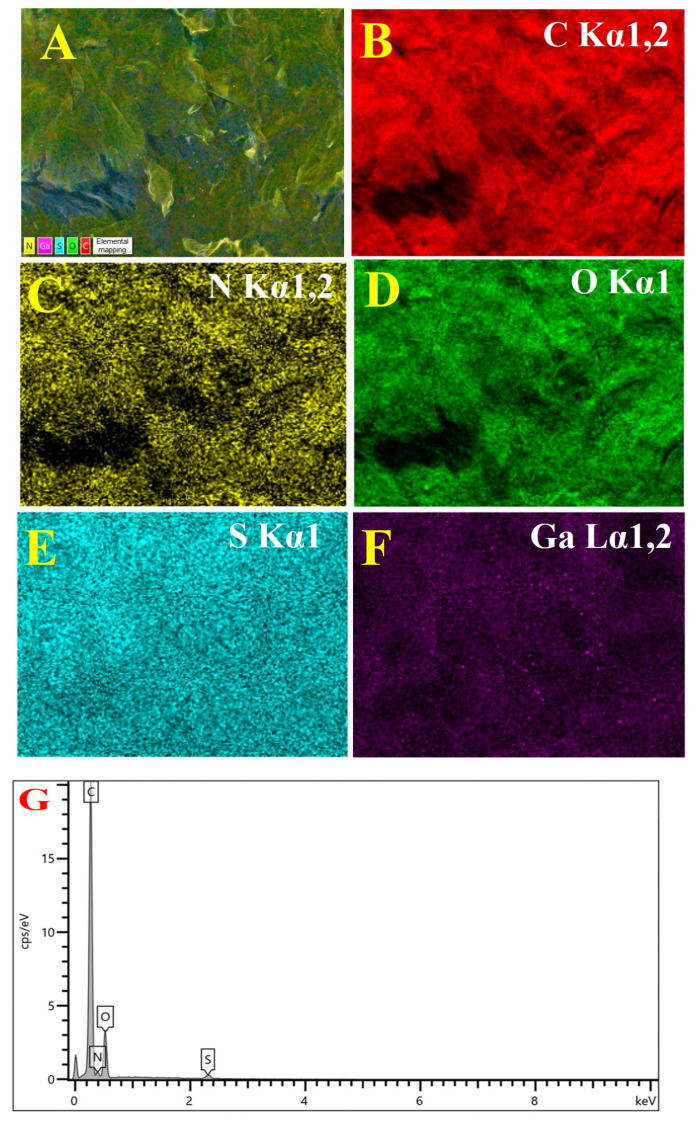
Elemental-mapping images of the GO-MTA composite post Ga^3+^ adsorption—(**A**) area tested, (**B**) C, (**C**) N, (**D**) O, (**E**) S, and (**F**) Ga^3+^—and (**G**) EDS of the composite.

The elemental distribution of the GO-MTA composite post Ga^3+^ adsorption was further elucidated (Figure 2A–F). Noticeably, N was uniformly distributed, along with C and O, on the surface of the GO-MTA composite (Figure 2B–D), while S was distributed almost evenly over the surface (Figure 2E). The distribution of Ga^3+^ was consistent with that of N, indicating that it had been successfully adsorbed onto the GO-MTA through strong cation–lone pair interactions (Figure 2C,F) [26]. According to the EDS analysis, 79.62 wt.% of C, 5.37 wt.% of N, 13.13 wt.% of O, and 4.62 wt.% of S were measured (Figure 2G).

#### 2.1.2. FT-IR Spectra

**Figure 3:**
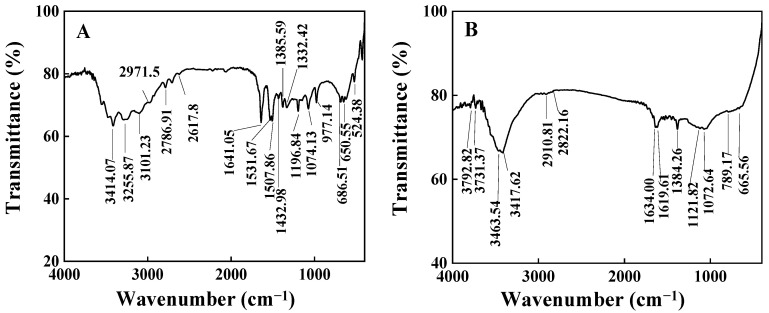
FT-IR spectra of the samples: (**A**) MTA and (**B**) GO-MTA composite.

The FT-IR spectra of the GO (Figure S1), MTA, and the GO-MTA composite were also recorded (Figure 3). For MTA (Figure 3A), the broad peaks observed at the wavenumbers of 3414.07, 3255.87, and 3101.23 cm^−1^ clearly confirmed the existence of hydrogen-bonding interactions and stretching vibrations of N-H [27]. The peaks at 2971.5 and 2786.91 cm^−1^ corresponded to the symmetric- and asymmetric-stretching vibrations of saturated C-H, respectively. A weak characteristic peak corresponding to S-H could be observed at 2617.8 cm^−1^, while two weak characteristic peaks corresponding to C=N could be detected at the wavenumbers of 1641.05 and 1531.67 cm^−1^. The peak at 686.51 cm^−1^ could be assigned to the ν(C-S)-stretching mode [28], and the characteristic peak at 1385.59 cm^−1^ confirmed the existence of -CH_3_. 

**Scheme 1:**
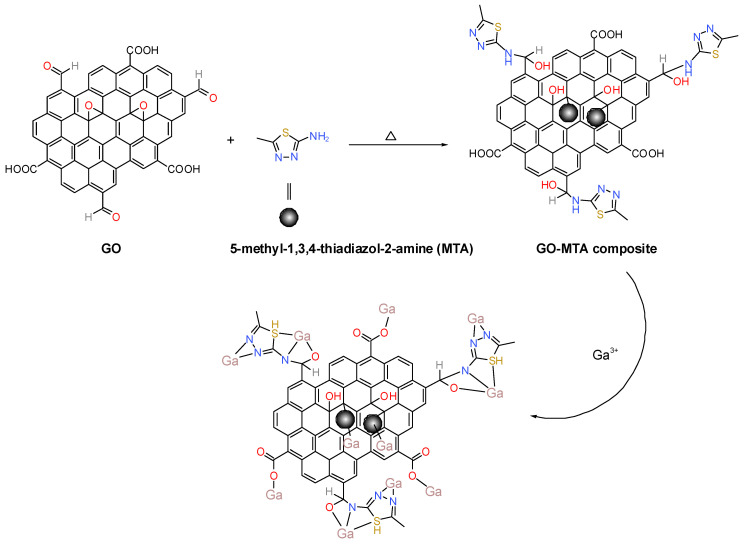
A schematic diagram of the functionalization of the GO-MTA composite and its Ga^3+^ adsorption mechanism.

In comparison, the GO-MTA composite possessed characteristic peaks corresponding to N-H (3417.6~3792.8 cm^−1^), -CH_3_ (2910.81, 2822.16, 1384.26 cm^−1^), C=N (1634 cm^−1^), and the skeleton vibrations of the benzene ring (1619.61 cm^−1^), confirming the successful attachment of MTA onto GO (Figure 3B). Due to the specific functional groups on the surface, adsorption might involve two processes: the release of protons from the surface -COOH groups, followed by Ga^3+^ coordination to the de-pronated sites, and the coordination interactions between Ga^3+^ and the N-N component (Scheme 1) [29]. The intrinsic Ga^3+^ adsorption mechanism of the GO-MTA composite was further determined using XPS and adsorption kinetic/isothermal models.

#### 2.1.3. XPS

**Figure 4:**
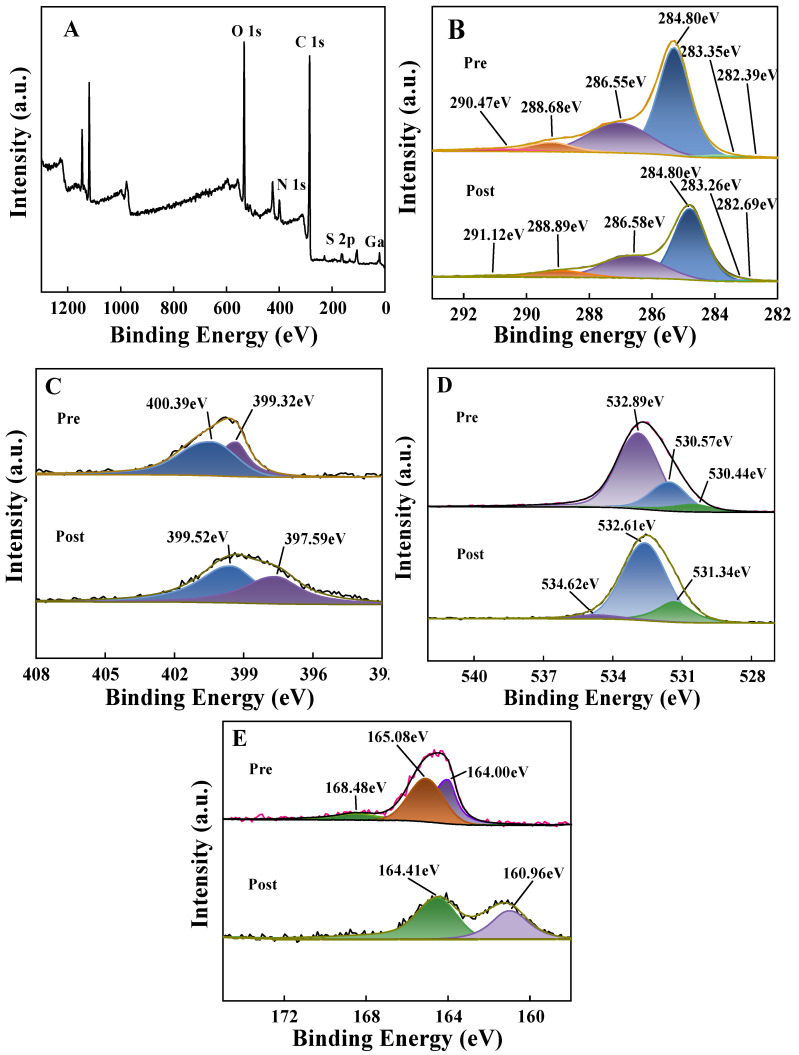
XPS spectra of the GO-MTA composite pre and post Ga^3+^ adsorption: (**A**) survey spectra and XPS peak-differentiation-imitating analyses of (**B**) C 1s, (**C**) N 1s, (**D**) O 1s, and (**E**) S 2p.

To reveal its composition and chemical state, XPS surface characterization of the GO-MTA composite pre and post Ga^3+^ adsorption was performed. The survey spectra of the GO-MTA composite indicated the presence of C, N, S, O, and Ga, while the XPS of the GO-MTA-Ga^3+^ revealed a new peak corresponding to the Ga (Figure 4A). Deconvolution of the C 1s region led to six peaks, located at 290.47, 288.68, 286.55, 284.80, 283.35, and 282.39 eV, corresponding to O=C-OH, O=C-H, C-O, C=C, C-S, and C=N bonds, respectively. After Ga^3+^ adsorption, peaks at 291.12, 288.89, 286.58, 284.80, 283.26, and 282.69 eV could be observed. The clear shifts in the binding energy of the O=C-OH and C=N bonds indicate that these two groups were involved in the adsorbent–adsorbate interactions (Figure 4B) [30].

Further deconvolution of the N 1s region showed two peaks located at 400.39 and 399.32 eV, which could be attributed to the C-N and -C=N bonds, respectively. After Ga^3+^ adsorption, the peaks of the GO-MTA-Ga^3+^ shifted to 399.52 and 397.59 eV, respectively, again confirming that the -C=N and C-N bonds might coordinate with Ga^3+^ due to EDA interactions (Figure 4C, Scheme 1) [31]. 

Previous studies have confirmed that the O-containing functional groups of oxidized carbon nanomaterials, such as the carboxyl (-COOH) and hydroxyl (-OH) groups, may participate in the adsorption process of positive metal cations [32]. In our study, the XPS peak-differentiation-imitating analyses of the O 1s region displayed three peaks located at 532.89, 530.57, and 530.44 eV, which could be assigned to the C-O-C, -O-C=O, and O-H bonds, respectively (Figure 4D). For GO-MTA-Ga^3+^, the abovementioned peaks shifted to 534.62, 532.61, and 531.34 eV, respectively, also guaranteeing the EDA interactions between the O-containing functional groups and Ga^3+^ (Scheme 1). 

To evaluate the possible interactions between the S-containing group and Ga^3+^, deconvolution of the S 2p region was performed (Figure 4E). Three peaks were located at 168.43, 165.08 and 164.00 eV, corresponding to the characteristic peaks of S 2p_3/2_ and S 2p_1/2_, respectively, shifting to 164.48 and 160.96 eV after Ga^3+^ adsorption [33]. These results suggested that the S-containing group might also be involved in the adsorption process (Scheme 1). As shown in Scheme 1, the simultaneous interaction of Ga^3+^ with the C-N, -OH, and C-S groups appeared to be infeasible or difficult. We therefore propose that the -C=N-N=C- groups might have dominated in the adsorption process, guaranteeing the comparatively high adsorption selectivity of the GO-MTA composite for Ga^3+^. The lone pairs of the two N atoms in heterocyclic -C=N-N=C- were located in the sp^2^ hybrid orbitals, making them relatively better electron donors [34].

### 2.2. Adsorption Experiments

#### 2.2.1. Adsorption Selectivity

**Figure 5:**
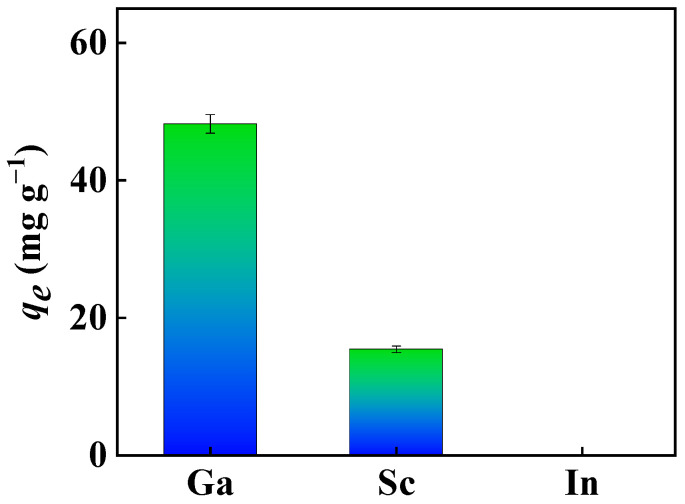
Adsorption properties of the GO-MTA composite for Ga^3+^, Sc^3+^, and In^3+^ (dosage of adsorbent = 5.0 mg, *V* = 20.0 mL, *C*_0_ = 50.0 mg L^−1^, *T* = 25 °C, and *t* = 180 min).

Different metal ion solutions including Ga^3+^, Sc^3+^, and In^3+^ were used to evaluate the adsorption properties of the GO-MTA composite. A total of 5.0 mg of the GO-MTA composite was added to a 50.0 mL conical bottle containing 20.0 mL of metal ion solution (50.0 mg L^−1^) and stirred at 25 °C for 180 min. The adsorption capacity of the GO-MTA composite for Ga^3+^, Sc^3+^, and In^3+^ was calculated to be 48.20, 15.41, and ~0 mg g^−1^, respectively (Figure 5), indicating that it possessed a relatively higher adsorption selectivity toward Ga^3+^ than the other two metal ions. We therefore propose that the N-N group was able to more strongly interact with Ga^3+^ than In^3+^ and Sc^3+^. As a result, Ga^3+^ was selected as a model ion to investigate the effects of conditional parameters on the adsorption process in the following experiments. 

#### 2.2.2. Effect of Contact Time and Adsorption Kinetics

**Figure 6:**
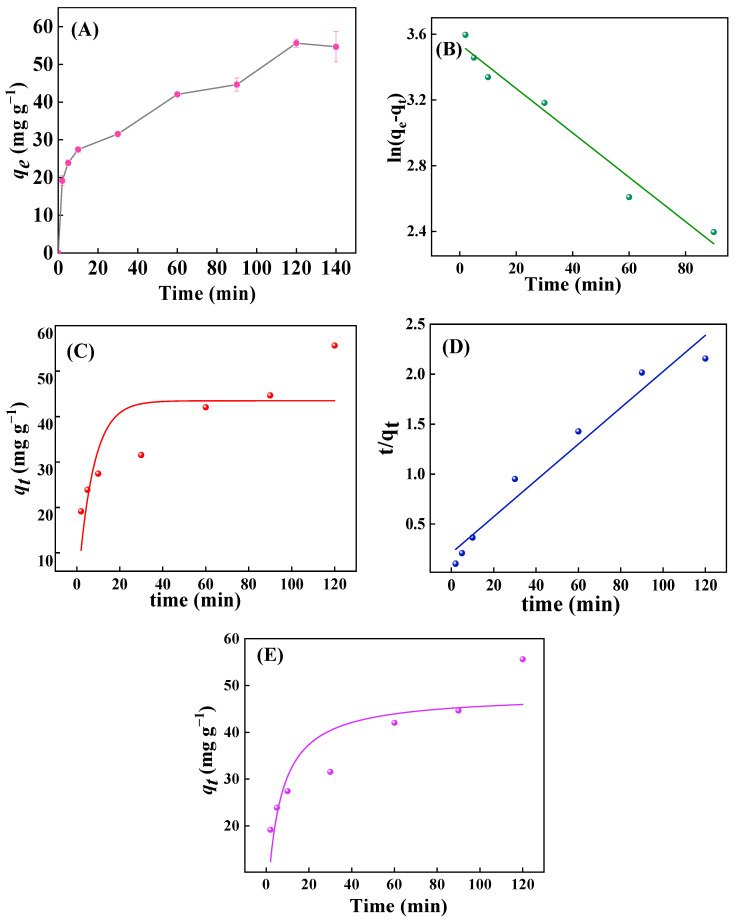
The adsorption capacity in MTA: (**A**) effect of the contact time (adsorbent dosage = 5.0 mg, *C*_0_ = 50.0 mg L^−1^, *T* = 298 K, and *V* = 20.0 mL; RSD = 0.14%~3.45%); (**B**) fitted using the linear pseudo-first-order kinetic model; (**C**) fitted using the nonlinear pseudo-first-order kinetic model; (**D**) fitted using the linear pseudo-second-order kinetic model; and (**E**) fitted using the nonlinear pseudo-second-order kinetic model.

The effect of the contact time on Ga^3+^ adsorption onto the GO-MTA composite was investigated (Figure 6A). We found that 120 min was enough to achieve adsorption equilibrium under the experimental conditions (adsorbent dosage = 5.0 mg, *C*_0_ = 50.0 mg L^−1^, and *T* = 298 K). Meanwhile, fast adsorption occurred in 15 min in the initial stage, followed by a gradually increased adsorption capacity. To achieve adsorption equilibrium, a contact time of 120 min was applied in the subsequent adsorption experiments.

The experimental data of the GO-MTA composite for Ga^3+^ at 298 K were fitted using different kinetic models (linear/nonlinear pseudo-first- and second-order models) (Equations (S1)–(S4)). As shown in Figure 6B–E and Table 1, the experimental data were distorted by the nonlinear kinetic forms, indicating that the linear ones should be primarily used to calculate the adsorption parameters [35]. The adsorption data could be well described by the linear pseudo-second-order kinetic model (*R*^2^ = 0.962), suggesting that the rate-limiting step may be chemical sorption or chemisorption through the sharing or exchange of electrons between the adsorbent and the adsorbate [36]. Importantly, the calculated *q_e_* value (55.066 mg g^−1^) from the plots of *t*/*q_t_* versus *t* was closer to the experimental result (55.60 mg g^−1^).

#### 2.2.3. Effect of Solution pH

**Figure 7:**
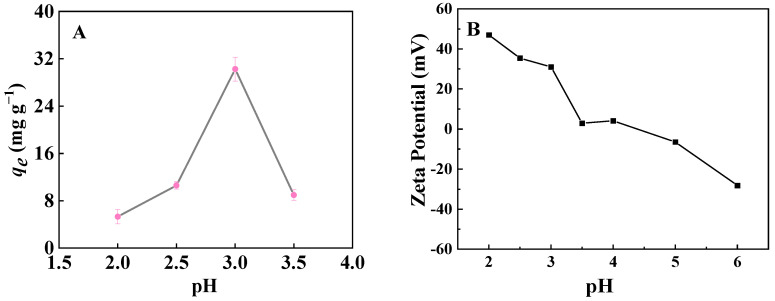
(**A**) Effects of the pH on the Ga^3+^ adsorption capacity of the GO-MTA composite (adsorbent dosage = 5.0 mg, *C*_0_ = 50.0 mg L^−1^, *T* = 298 K, *V* = 20.0 mL, and *t* = 120 min; RSD = 0.25%~3.04%). (**B**) Zeta potential of the GO-MTA composite in the pH range of 2.0–6.0.

The initial pH value of a solution is one of the most important parameters able to affect metal cation adsorption. Figure 7A reflects the relationship between the adsorption capacity of the GO-MTA composite and the initial pH value of the solution (*t* = 120 min, adsorbent dosage = 5.0 mg, *C*_0_ = 50.0 mg L^−1^, and *T* = 298 K). It is clear that its adsorption capacity increases with an increase in the initial pH in the range of 2.0–3.0. The active sites of the N-containing groups on the surface of the GO-MTA composite are protonated under strongly acidic conditions, negatively influencing adsorption due to Ga^3+^ also being positively charged [37]. That is, the active sites are readily occupied by H^+^ rather than Ga^3+^. It is well known that Ga^3+^ is precipitated at higher pH values [38], whereby sedimentation rather than adsorption occurs, resulting in decreased adsorption capacities. The zeta potentials of the GO-MTA composite in the pH range of 2.0–6.0 were measured (Figure 7B). A large number of N-N groups were present on the surface of the GO-MTA composite. Noticeably, its zeta potentials in the tested pH range decreased gradually and then turned negative at pH = 5.0. The maximum adsorption capacity at pH = 3.0 indicated that the O-H, C-N, and S-C groups were positively charged at low pH values and that the electrostatic repulsions between the adsorbent and the adsorbate had a negative effect on adsorption. The complexation interactions between the N-N groups of the GO-MTA composite and Ga^3+^ mainly contributed to the effective adsorption, and the Ga^3+^ adsorption capacity of the composite gradually increased as the solution’s pH increased.

#### 2.2.4. Adsorption Isotherms

Adsorption isotherms can be established when dynamic adsorption equilibrium is achieved. The adsorption progress is usually affected by experimental factors, including the solution’s pH, the contact temperature, the initial adsorbate concentration, and especially, the nature of the adsorbent. Optimizing adsorption processes by rationally changing the conditional parameters is of great significance. Most often, the adsorption isotherm could be obtained by changing the initial adsorbate concentration and contact temperature whilst keeping the other conditions unchanged. In this work, different types of isothermal models (linear/nonlinear Langmuir and Freundlich isothermal models) were employed to diagnose the nature of Ga^3+^ adsorption by the GO-MTA composite (Equations (S5)–(S8)).

**Figure 8:**
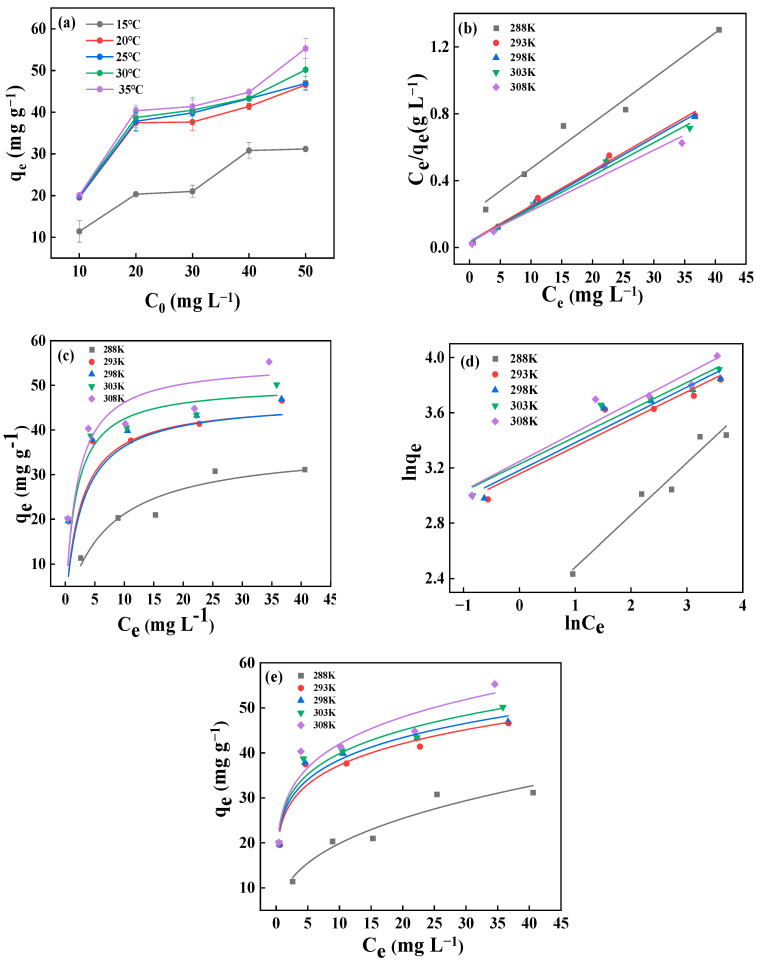
Effects of the contact time on the GO-MTA composite’s Ga^3+^ adsorption capacity: (**a**) effect of the initial concentration and temperature on the adsorption capacity of the GO-MTA (adsorbent dosage = 5.0 mg, *C*_0_ = 50.0 mg L^−1^, *T* = 298 K, *V* = 20.0 mL, *t* = 120 min, and pH = 3.0; RSD = 0.32%~2.12%); (**b**) fitted curve of the linear Langmuir isothermal model; (**c**) fitted curve of the nonlinear Langmuir isothermal model; (**d**) fitted curve of the linear Freundlich isothermal model; and (**e**) fitted curve of the nonlinear Freundlich isothermal model.

Noticeably, the experimental data of the GO-MTA composite for Ga^3+^ could be better described by the linear Langmuir isothermal model. As can be seen from Figure 8a–e and Table 2, the linear regression of *C_e_*/*q_e_* vs. *C_e_* provides the best fit for the equilibrium data (*R*^2^ = 0.972–0.998). The estimated adsorption capacity *q_m_* is 55.556 mg g^−1^ at 308 K, which is much closer to the experimental data. In addition, the values of 0 < *k_L_* < 1 suggest that adsorption is an endothermic process, which is also consistent with the experimental data (Figure 8a) [39]. The interfacial monolayer’s capture efficiency for the adsorbate was also confirmed by the linear Langmuir isothermal model. However, cooperative adsorption caused primarily by attractive interactions between the monolayer and the dissolved adsorbate on a heterogeneous surface might occur.

#### 2.2.5. Adsorption Thermodynamics

Figure S2 shows the experimental data and the fitted curve of Ink_d_ versus 1/T calculated from the Van’t Hoff plots of the GO-MTA composite for Ga^3+^ adsorption. We can observe that the adsorption process is temperature-dependent and that an increase in the contact temperature results in a corresponding increase in the Ga^3+^ adsorption capacity of the GO-MTA composite. Noticeably, the results are in accordance with the adsorption isotherms fitted using the linear Langmuir isothermal model.

The standard free-energy change, *ΔG^o^*, was calculated. The average standard enthalpy change (*ΔH^o^*) was calculated from the Van’t Hoff equation. The thermodynamic parameters are listed in Table S1. Noticeably, the *ΔG^o^* values at all the tested temperatures and initial Ga^3+^ concentrations are negative, suggesting that Ga^3+^ adsorption by the GO-MTA composite is spontaneous. The *ΔG^o^* values become more negative as the contact temperature increases, indicating that higher temperatures benefit adsorption. Interestingly, the *ΔG^o^* values become less negative with increasing initial Ga^3+^ concentrations, implying that the presence of a specific external surface dominates physical adsorption. As the initial Ga^3+^ concentration increases, chemical adsorption due to the coordination effects between the adsorbate and the N-, O-, and S-containing functional groups of the GO-MTA composite dominates the process, as further verified by the positive *ΔH^o^* values calculated. That is, Ga^3+^ adsorption by the GO-MTA composite is an endothermic process, as supported by the increased Ga^3+^ adsorption with an increase in the contact temperature (Table S1).

Finally, the positive *ΔS^o^* values could be attributed to the transfer of the Ga^3+^ ion in the solutions caused by the interactions between Ga^3+^ and the functional groups of the GO-MTA composite [40].

### 2.3. Comparison of Ga^3+^ Adsorption with Previously Reported Adsorbents

The Ga^3+^ adsorption capacity of the newly developed GO-MTA composite was compared with that of various adsorbents previously reported in the literature (Table 3). Noticeably, in some cases, the experimental data from this investigation exhibited some advantages over previous data. The adsorbents’ adsorption capacity varied to a certain extent, depending on the individual physicochemical characteristics, the substrate, and the surface functional groups, as well as the initial adsorbate concentration. Importantly, the GO-MTA composite was relatively easier to fabricate and possessed a higher adsorption selectivity for Ga^3+^. To rationally design and develop a practically applicable adsorbent with specific adsorption selectivity toward Ga^3+^, the following experiments were carried out. These experiments could be used as a useful reference for follow-up studies. 

### 2.4. Desorption and Reutilization

**Figure 9:**
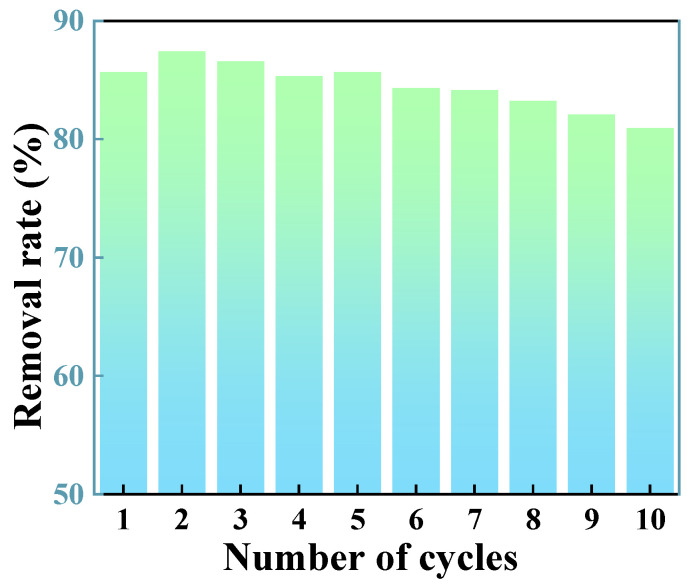
The reusability of the GO-MTA composite.

From the perspective of efficient resource use for sustainable development, the reutilization of a newly developed adsorbent should be evaluated. In this study, diluted nitric acid (HNO_3_, 1.0 mol L^−1^) was used as the eluent during the desorption process to reveal the GO-MTA composite’s reusability. After Ga^3+^ adsorption at room temperature for 6 h, the adsorbent was stirred, obtaining an elution rate of ~90%. The GO-MTA composite’s adsorption efficiency during 10 adsorption–desorption cycles was investigated, showing that the removal rate remained above ~80% even after all the cycles (Figure 9). Generally, the GO-MTA composite possesses a relatively higher stability and may be used for practical applications.

### 2.5. Adsorption Properties of the GO-MTA Composite in a Mixed Solution

The GO-MTA composite (50.0 mg) was applied to a mixed solution (20.0 mL) containing Sc^3+^ (10.0 mg L^−1^) and Ga^3+^ (10.0 mg L^−1^) in a conical flask (50.0 mL) at 35 °C for 180 min. Relatively lower adsorption capacities of 0.45 mg mL^−1^ and 15.76, respectively, were determined. Noticeably, the GO-MTA composite exhibited a relatively higher adsorption selectivity toward Ga^3+^ even in a mixed solution, suggesting its potential applications in real samples.

## 3. Experimental Details 

### 3.1. Fabrication of the GO Nanosheets

The GO suspension was easily prepared according to the modified Hummer method [47,48]. In brief, flake graphite (0.60 g) was evenly mixed with KMnO_4_ powder (3.00 g), then transferred into a round-bottom bottle (500.0 mL) placed in an ice-water bath. Under constant stirring, a mixture of H_3_PO_4_ (85%, 8.0 mL) and H_2_SO_4_ (98%, 72.0 mL) was slowly added to the reaction. Once the dispersion had gradually turned a bottle-green color, it was heated to 50 °C and reacted for 12 h until it turned purple–red. Once the mixture had naturally cooled to room temperature, excess H_2_O_2_ was added until it turned bright yellow, with no bubbles. The mixture was washed repeatedly with a certain amount of HCl (1.0 mol L^−1^) and ultrapure water, and it was separated by centrifugation until the supernatant’s pH was close to being neutral. Finally, the black solid residual was carefully collected, dispersed in a certain amount of ultrapure water, and then freeze-dried at −50 °C for 48 h to obtain GO nanosheets.

### 3.2. Fabrication of the GO-MTA Composite

GO (500.0 mg) was dispersed in 50.0 mL of ultrapure water in a 100.0 mL round-bottom flask and then ultrasonically treated to obtain an evenly mixed GO dispersion. Afterwards, 1.0 mL of CH_3_COOH (99.5 wt.%) and 2.0 g of MTA were added to the reaction, followed by heating the mixture to 110 °C in an oil bath for 8 h. After the reaction, the mixture was naturally cooled to room temperature, diluted with a certain amount of ultrapure water, and then filtered and washed 3 times with ethanol and ultrapure water until the filtrate had turned neutral. The residual solid on the filter paper was collected, then dispersed in a certain amount of ultrapure water, and finally, freeze-dried at −50 °C for 36 h to obtain the GO-MTA composite. 

### 3.3. Batch Adsorption Experiments

The effects of the contact time, initial pH of the solution, initial Ga^3+^ concentration, and contact temperature on the GO-MTA composite’s Ga^3+^ adsorption performance were investigated. Ga^3+^ solutions were prepared by dissolving a certain amount of Ga(NO_3_)_3_∙H_2_O in ultrapure water and then transferred into a volumetric flask and diluted to a certain volume. A total of 5.0 mg of the GO-MTA composite was mixed with 20.0 mL of Ga(NO_3_)_3_ solution in a 50.0 mL conical flask and continuously stirred at a certain temperature (15 °C, 20 °C, 25 °C, 30 °C, and 35 °C) for a certain time (0–180 min). Three parallel experiments were performed, and the adsorption data were calculated using Equation (1) to obtain the equilibrium adsorption capacity (*q_e_*; mg g^−1^), while the removal rate (*R*, %) was calculated using Equation (2):

(1) $$q_{e}=\frac{(C_{0}-C_{e})\cdot V}{m}$$

(2) $$R=\frac{C_{0}-C_{e}}{C_{0}}\times 100\%$$

where *C*_0_ (mg L^−1^) represents the initial Ga^3+^ concentration; *C_e_* (mg L^−1^) represents the equilibrium Ga^3+^ concentration; and *V* (L) and *m* (g) represent the volume of the Ga^3+^ solution and the mass of the GO-MTA composite, respectively.

### 3.4. Sample Characterization

The morphology and elemental distributions of the GO-MTA composite pre and post Ga^3+^ adsorption were verified using a field emission scanning electron microscope (FE-SEM; JSM-7900F, JEOL Corp.; Tokyo, Japan) equipped with an energy-dispersive X-ray spectroscope (EDS) at an accelerating voltage of 10 kV. The Fourier transform infrared (FTIR) spectra were recorded on a Shimadzu FTIR (IR Prestige-21; Shimadzu, Ltd., Tokyo, Japan) spectrophotometer in the wavenumber range of 4000–400 cm^−1^ and at a resolution of 4 cm^−1^ to detect the surface functional groups of the GO, MTA, and GO-MTA composite. The composition and chemical states of the GO-MTA composite pre and post Ga^3+^ adsorption were characterized using an X-ray photoelectron spectroscope (XPS) on a Perkin Elmer PHI 5000 C ESCA instrument (Perkin Elmer Co.; Eden Prairie, MN, USA) using Al Kα radiation (1486.6 eV) with a detection angle of 54° and a cathode voltage of 14 kV, operated at 250 W. The metal ion concentration was determined via an inductively coupled plasma–optical emission spectrometer (ICP-OES; IRIS Intrepid II XSP; Thermo Electron Corporation, Waltham, MA, USA). 

## 4. Conclusions

In this study, a highly selective GO-MTA composite with a specific N-containing functional group was fabricated through the nucleophilic addition of MTA and GO. This GO-MTA composite exhibits a stacked lamellar structure. Due to the surface N-, O-, and S-containing functional groups, the GO-MTA composite exhibits a relatively higher adsorption capacity and selectivity for Ga^3+^ compared to Sc^3+^ and In^3+^. In our experiments, at an initial concentration of 50.0 mg L^−1^, a contact temperature of 35 °C, a contact time of 120 min, and an adsorbent dosage of 5.0 mg, a high Ga^3+^ adsorption capacity of 55.6 mg g^−1^ could be observed, and the adsorption process could be best described by the linear Langmuir isothermal model (*R*^2^ = 0.972–0.997) and the linear pseudo-second-order kinetic model (*R*^2^ = 0.962). Ga^3+^ adsorption by the GO-MTA composite under acidic conditions was mainly dominated by the coordination interactions of the adsorbate with the adsorbent’s -C=N-N=C- functional group. Elution rates of ~90% could be obtained using HNO_3_, and a removal rate above ~80% could be found even after 10 adsorption–desorption cycles. Application of the GO-MTA composite in a mixed solution containing Sc^3+^ and Ga^3+^ confirmed its excellent adsorption selectivity. These results suggest that the GO-MTA composite could be implemented as a potential adsorbent for Ga^3+^ separation/enrichment processes in aqueous solutions.

## Figures and Tables

**Table 1:** Adsorption kinetic parameters for adsorption by MTA.

Adsorption Kinetic Model	*R* ^2^	*k*_1_ (min^−1^)	*k*_2_ (min^−1^)	*q_e_* (mg g^−1^)
Linear pseudo-first-order	0.971	0.013	-	9.622
Nonlinear pseudo-first-order	0.621	0.139	-	43.470
Linear pseudo-second-order	0.962	-	0.0015	55.066
Nonlinear pseudo-second-order	0.769	-	0.0036	48.156

**Table 2:** Isothermal parameters for adsorption onto the GO-MTA composite.

	*T* (K)	Langmuir Model			Freundlich Model		
		*R* ^2^	*k_L_*	*q_m_* (mg g^−1^)	*R* ^2^	*k_F_*	n
Linear	288	0.972	0.134	37.037	0.956	5.717	2.646
	293	0.993	0.583	47.619	0.922	8.579	5.039
	298	0.997	0.703	47.847	0.931	8.468	4.965
	303	0.990	0.576	52.631	0.942	8.771	5.062
	308	0.978	0.474	55.556	0.931	8.824	4.727
Nonlinear	288	0.916	0.137	36.567	0.931	8.731	2.803
	293	0.521	0.377	46.560	0.913	24.877	5.706
	298	0.406	0.343	46.893	0.924	25.730	5.734
	303	0.725	0.553	50.160	0.934	26.510	5.656
	308	0.715	0.507	55.260	0.914	26.924	5.188

Notes: The units of *k_L_* and *k_F_* are L mg^−1^ and mg L^1/n^ g^−1^ mg^−1/n^, respectively.

**Table 3:** Ga^3+^ adsorption comparison with previously reported adsorbents.

Adsorbent	*q_e_* (mg g^−1^)	*C*_0_ (mg L^−1^)	*T* (°C)	Reference
γ-Al_2_O_3_	Not provided	210	25	[5]
PAN NFs	39.80	200	30	[21]
GO-AHTZT	34.72	50	35	[23]
A-PSD resin	14.67	200	25	[29]
GO/UiO-66-NH_2_	661.67	400	40	[41]
RCONHOH	27.65	500	60	[42]
NHPA	120.48	300	RT	[43]
CNT/UiO-66-NH_2_	925.44	400	40	[44]
Catechol-modified resin	28.71	60	RT	[45]
KIT-6@CA-PEI	88.65	20	40	[46]
GO-MTA	55.6	50	35	This work

Notes: A-PSD: amidoxime-grafted polyacrylonitrile-styrene–divinylbenzene; PAN NFs: polyacrylonitrile nanofiber membranes; RCONHOH: polyacrylate-divinylbenzene hydroxamic resins; NHPA: nitrogen-rich functional group biomass carbon aerogel; CNT: carbon nanotube; RT: room temperature; and KIT-6@CA-PEI: mesoporous silica@x-catechol/polyethyleneimine.

## Data Availability

The original contributions presented in this study are included in this article/the Supplementary Materials. Further inquiries can be directed to the corresponding author.

## Supplementary Materials

The following supporting information can be downloaded at https://www.mdpi.com/article/10.3390/molecules29163768/s1: Figure S1: FT-IR spectra of GO; Figure S2: Experimental data and the fitted curve of Ink_d_ versus 1/T calculated from the Van’t Hoff plots of the GO-MTA composite for Ga^3+^ with different concentrations—(A) 10 mg L^−1^; (B) 20 mg L^−1^; (C) 30 mg L^−1^; (D) 40 mg L^−1^; and(E) 50 mg L^−1^; and Table S1: Adsorption thermodynamic parameters of the GO-MTA composite for Ga^3+^.
