# Supplementary material for: Graphene Oxide Covalently Functionalized with 5-Methyl-1,3,4-thiadiazol-2-amine for pH-Sensitive Ga3+ Recovery in Aqueous Solutions
Source: Molecules. 2024 Aug 9;29(16):3768. doi: 10.3390/molecules29163768 (PMC11357510; doi:10.3390/molecules29163768)
Supplement: Supplementary file 1 [file molecules-29-03768-s001.zip › molecules-3079206-supplementary.pdf]

Supporting Information

Graphene Oxide Covalently Functionalized with 5-Methyl-1,3,4-thiadiazol-2-amine for pH-Sensitive Ga^{3+} Recovery in Aqueous Solutions

Xi Zhu, Yong Guo *, Baozhan Zheng

College of Chemistry，Sichuan University, Chengdu 610065, China

To whom correspondence should be addressed.

* Y Guo, E-mail: guoy@scu.edu.cn;

1. Reagents and materials

Potassium permanganate ($KMnO_4$) and phosphoric acid (H_3PO_4, 85 wt.%) were bought from Sinopharm Group Chemical Reagent Co., Ltd. (Shanghai, China); Hydrochloric acid (HCl, 37 wt.%), sodium hydroxide (NaOH; AR, 97 wt.%), and concentrated sulfuric acid (H_2SO_4, 98 wt.%) were purchased from Chengdu Cologne Chemical Co. Ltd. (Chengdu, China); Flake graphite (80-90.5 wt.%) was supplied by Qingdao Braide Graphite Co., Ltd (Qingdao, China); 5-methyl-1,3,4-thiadiazol-2-amine (MTA, $C_3H_5N_3S$, 99 wt.%) , acetic acid (CH_3COOH, 99.5 wt.%) were purchased from Shanghai Aladdin Bio-Chem Technology Co., Ltd. (Shanghai, China); Hydrogen peroxide (H_2O_2, 30.0 wt.%) was bought from Shanghai Wokai Biotechnology Co., Ltd. (Shanghai, China); Indium(III) chloride ($InCl_3$, 99.99%), scandium(III) nitrate hydrate （$Sc(NO_3)_3·H_2O$; 99.9 wt.%） and gallium(III) nitrate hydrate ($Ga(NO_3)_3·H_2O$; 99.99 wt.%) were bought from Shanghai Run-Biotech Co., Ltd. (Shanghai, China); All chemicals were of analytical grade and used without further purification. Ultrapure water with a resistivity of 18.2 MΩ cm^{-1} was produced from a Millipore Milli-Q water purification system (Millipore Trading Co., Ltd.; Shanghai, China).

2. FT-IR spectra of GO

As shown in **Figure S1**, the peaks at 3391.8 and 1721.2 cm^{-1} can be attributed to the stretching vibrations of the hydroxyl (–OH) and C=O groups of GO, respectively. In addition, the peak at 1622.1 cm^{-1} can be assigned to the C=O stretching vibrations of carboxyl groups, and the peak at 1567.9 corresponds to the aromatic skeletal vibrations, while the peaks at 1229.8 and 1037.5 cm^{-1} belong the C–O and C-C stretching vibrations, respectively.

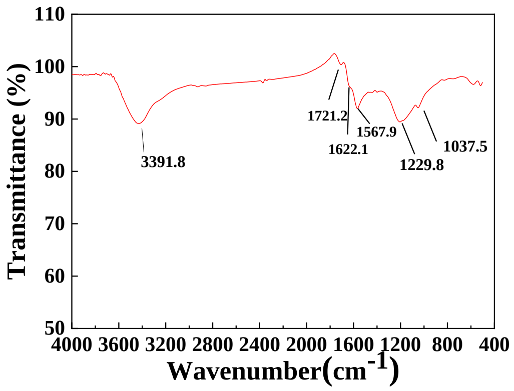

Figure S1 FT-IR spectra of GO.

3. Adsorption kinetic models

The linear pseudo-first-order rate and the linear pseudo-second-order rate equation are used to simulate the adsorption kinetics (**Equations (S1) and (S2)**):

Linear pseudo-first-order model: $\ln(q_e - q_t) = \ln q_e - k_1 t$ (S1)

Nonlinear pseudo-first-order model: $q_t = q_e(1\text{-}e^{-k_1 t})$ (S2)

Linear pseudo-second-order model: $\frac{t}{q_t} = \frac{1}{k_2 q_e^2} + \frac{t}{q_e}$ (S3)

Nonlinear pseudo-second-order model: $q_t = \frac{k_2 {q_e}^2 t}{1 + k_2 q_e t}$ (S4)

where q_e (mg g^{-1}) and q_t (mg g^{-1}) are the amounts of Ga^{3+} adsorbed by GO-MTA

composite at equilibrium state and at contact time t (min), respectively; k_1 (min^{-1}) and k_2 (g mg^{-1} min^{-1}) are the specific adsorption rate constant of the pseudo-first-order model and the pseudo-second-order model, respectively.

4. Adsorption isothermal models

The linear/nonlinear Langmuir isothermal model and the linear/nonlinear Freundlich isothermal model are used to simulate the adsorption isotherms (**Equations (S5)–(S8)**):

Linear Langmuir isothermal model: $$\frac{C_e}{q_e} = \frac{C_e}{q_m} + \frac{1}{q_m k_L} \quad \text{(S5)}$$

Nonlinear Langmuir isothermal model: $$q_e = \frac{q_m k_L C_e}{1 + k_L C_e} \quad \text{(S6)}$$

Linear Freundlich isothermal model: $$\ln q_e = \ln k_F + \frac{1}{n} \ln C_e \quad \text{(S7)}$$

Nonlinear Freundlich isothermal model: $$q_e = k_F C_e^{\frac{1}{n}} \quad \text{(S8)}$$

where C_e (mg L^{-1}) is the equilibrium concentration of Ga^{3+}; q_e (mg g^{-1}) is the amount of Ga^{3+} adsorbed by GO-MTA composite at equilibrium state; q_m (mg g^{-1}) is the monolayer adsorption capacity; k_L (L mg^{-1}) is the Langmuir equilibrium constant; The two Freundlich constants, n represents the relative advantage of adsorption process, k_F (mg $L^{1/n}$ g^{-1} $mg^{-1/n}$) is defined as adsorption or distribution coefficient which corresponds to the amount of Ga^{3+} adsorbed on GO-MTA composie at unit equilibrium concentration. The slope 1/n ranging between 0 and 1 could be used as a measure of adsorption intensity or surface heterogeneity, and a value close to zero suggests a more heterogeneous surface.

5. Adsorption thermodynamics

The change in the enthalpy (ΔH^o, kJ mol^{-1}), the change in the entropy (ΔS^o, J K^{-1}

mol^{-1}) and the change in the Gibbs free energy (ΔG^o, kJ mol^{-1}) can be calculated by **Equations (S9)–(S11)**.

$$k_d = \frac{C_0 - C_e}{C_e} \times \frac{V}{m} \quad \text{(S9)}$$

$$\ln k_d = \frac{-\Delta G^o}{RT} = \frac{\Delta S^o}{R} - \frac{\Delta H^o}{RT} \quad \text{(S10)}$$

$$\Delta G^o = \Delta H^o - T\Delta S^o \quad \text{(S11)}$$

where k_d (L mg^{-1}) represents the thermodynamic equilibrium constant; V (L) and m (g) are defined as the volume of Ga^{3+} solution and the mass of GO-MTA composite, respectively; T (K) represents the absolute temperature; R (8.314 J mol^{-1} K^{-1}) represents the ideal gas constant.

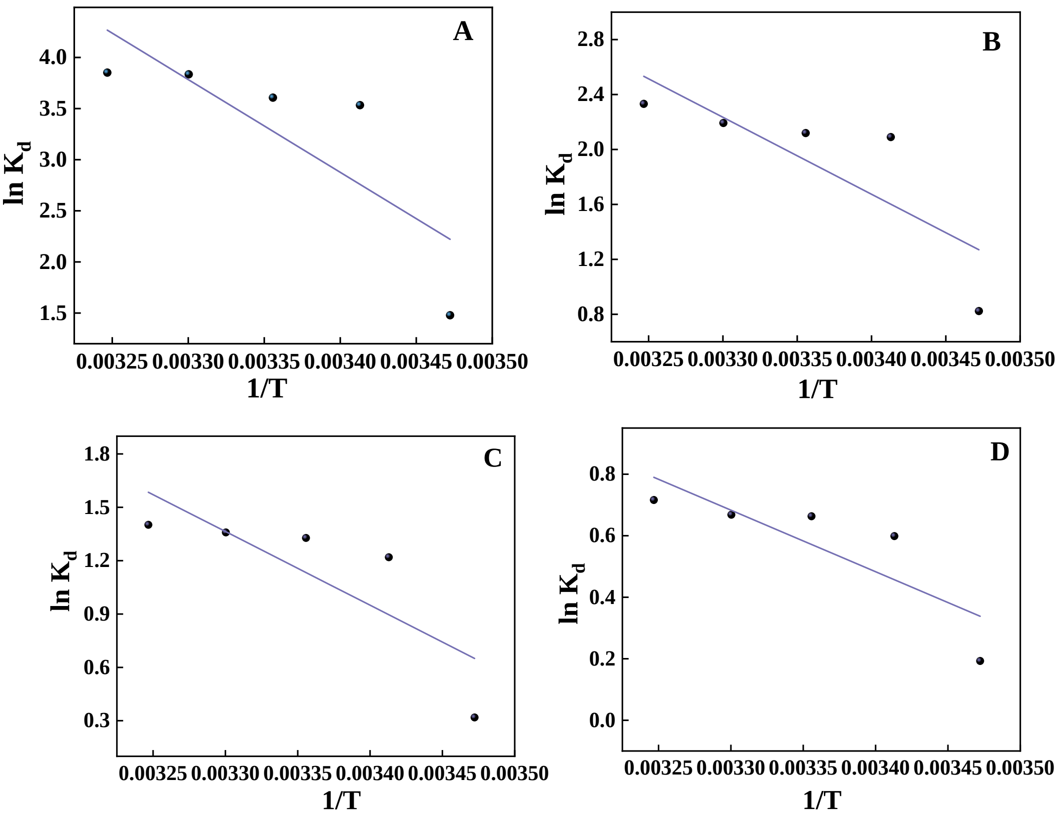

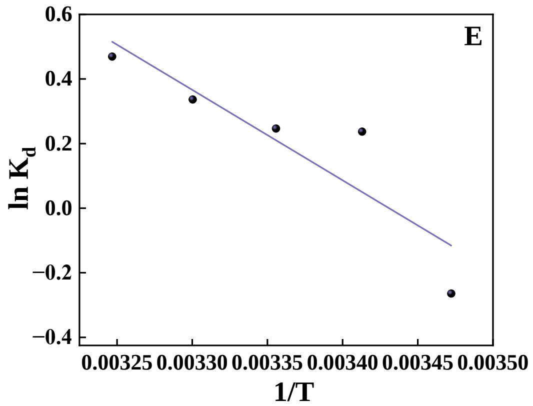

Figure S2 Experimental data and the fitted curve of *Ink$_d$* versus *1/T* calculated from Van't Hoff plots of GO-MTA composite for Ga^{3+} with different concentration: (A) 10 mg L^{-1}; (B) 20 mg L^{-1}; (C) 30 mg L^{-1}; (D) 40 mg L^{-1}; (E) 50 mg L^{-1}.

Table S1 Adsorption thermodynamic parameters of GO-MTA composite for Ga^{3+}.

C_0（mg L^{-1}）	T (K)	$\Delta G^{\ominus}$ (kJ mol^{-1})	R^2	$\Delta H^{\ominus}$ (kJ mol^{-1})	$\Delta S^{\ominus}$ (J mol^{-1} K^{-1})
10	288	-5.318	0.646	75.45	280.43
	293	-6.721			
	298	-8.123			
	303	-9.525			
	308	-10.927			
20	288	-3.040	0.658	46.57	172.24
	293	-3.040			
	298	-4.762			
	303	-5.623			
	308	-6.485			
30	288	-1.557	0.656	34.45	125.03
	293	-2.182			
	298	-2.808			
	303	-3.433			
	308	-4.058			
40	288	-0.809	0.698	16.66	60.66
	293	-1.113			
	298	-1.416			
	303	-1.719			
	308	-2.023			
50	288	0.277	0.802	23.27	79.82
	293	-0.122			
	298	-0.521			
	303	-0.920			
	308	-1.319			
